# Intelligent Medical IoT-Enabled Automated Microscopic Image Diagnosis of Acute Blood Cancers

**DOI:** 10.3390/s22062348

**Published:** 2022-03-18

**Authors:** Mohamed Esmail Karar, Bandar Alotaibi, Munif Alotaibi

**Affiliations:** 1 College of Computing and Information Technology, Shaqra University, P.O. Box 33, Shaqra 11961, Saudi Arabia; mkarar@su.edu.sa; 2 Faculty of Electronic Engineering, Menoufia University, Menouf 32952, Egypt; 3 Department of Information Technology, University of Tabuk, Tabuk 47731, Saudi Arabia; b-alotaibi@ut.edu.sa; 4 Sensor Networks and Cellular Systems (SNCS) Research Center, University of Tabuk, Tabuk 47731, Saudi Arabia

**Keywords:** acute leukemia, generative adversarial networks, computer-aided diagnosis, internet of medical things, wireless microscopic imaging

## Abstract

Blood cancer, or leukemia, has a negative impact on the blood and/or bone marrow of children and adults. Acute lymphocytic leukemia (ALL) and acute myeloid leukemia (AML) are two sub-types of acute leukemia. The Internet of Medical Things (IoMT) and artificial intelligence have allowed for the development of advanced technologies to assist in recently introduced medical procedures. Hence, in this paper, we propose a new intelligent IoMT framework for the automated classification of acute leukemias using microscopic blood images. The workflow of our proposed framework includes three main stages, as follows. First, blood samples are collected by wireless digital microscopy and sent to a cloud server. Second, the cloud server carries out automatic identification of the blood conditions—either leukemias or healthy—utilizing our developed generative adversarial network (GAN) classifier. Finally, the classification results are sent to a hematologist for medical approval. The developed GAN classifier was successfully evaluated on two public data sets: ALL-IDB and ASH image bank. It achieved the best accuracy scores of 98.67% for binary classification (ALL or healthy) and 95.5% for multi-class classification (ALL, AML, and normal blood cells), when compared with existing state-of-the-art methods. The results of this study demonstrate the feasibility of our proposed IoMT framework for automated diagnosis of acute leukemia tests. Clinical realization of this blood diagnosis system is our future work.

## 1. Introduction

Blood cancers, named leukemias [1], can be classified as aggressive illnesses. This illness is correlated with the white blood cells (WBCs) or leukocytes; thus, the human body can be adversely affected by this disease, the blood and bone marrow in particular. The prevalence of blood cancers has been increasing annually, due to genetic factors and/or environmental factors such as the presence of chemicals, among other unknown factors [2]. Their incidence and mortality rates have been ranked as 15th and 10th for all malignant cases, respectively [3,4]. Acute leukemia can be categorized into two main classes: myeloid and lymphoid [5]. Acute lymphocytic leukemia (ALL) is the most common leukemia in children, while acute myeloid leukemia (AML) is the most common malignant blood cancer in adults [1]. Male patients are the predominant focus of cases of both ALL and AML.

The traditional method used to detect leukemia is microscopic blood tests [6]. Another well-known technique to detect leukemia is through blood smear analysis. A further mechanism utilized to diagnose leukemia is interventional radiology. Other methods can also be used to detect leukemia, such as molecular cytogenetics and array-based comparative genomic hybridization (ACGH). However, all of these techniques are time-consuming and relatively expensive. In addition, the experience of the hematologist plays a major role in accomplishing diagnostic procedures on blood images. Therefore, the application of medical image analysis and computer-aided diagnosis (CAD) systems can provide the powerful capabilities of automatic detection and classification of leukemia, in order to provide support to medical staff [7,8].

It is of paramount significance for hematologists to detect leukemia and to distinguish between its sub-classes, to both avert medical risks and select the right medical treatment. The early detection of leukemia can be accomplished through the use of artificial intelligence (AI) techniques utilizing blood cell images (e.g., blood smears) [9]. Various CAD techniques utilizing machine learning and deep learning algorithms for the quantitative analysis of peripheral blood samples have been proposed [7]. Nevertheless, these techniques suffer from some shortcomings related to low accuracy, inefficiency, and learning process issues, affected by the availability of high computational resources.

To overcome the above limitations, the Internet of Things (IoT) paradigm presents advanced key solutions to establish new and accurate diagnosis systems for microscopic blood images, as is described in this study. The IoT has been deployed in diverse areas, such as smart cities [10,11], vehicular communications [12], smart ecosystems [13], smart farming and precision agriculture [14,15,16], and smart campuses [17,18]. Consequently, the Internet of Medical Things (IoMT) or smart healthcare [19,20] has been proposed for the improvement of quality of life for patients. IoMT, in a simple form, consists of both Wi-Fi-based smart medical gadgets and smart applications. These smart things should be connected, through computer networks, to IT health systems [21,22]. Sensors or other computing resources are integrated with the smart medical devices and spread throughout homes, clinics, communities, and hospitals [23]. These smart medical devices can collect and transmit data to cloud platforms for further processing and analysis [24]. In general, the IoMT paradigm consists of long-distance care for people with long-term diseases, patient medication monitoring, hospitalized patient tracking, and information supply to healthcare providers [25]. Therefore, with the help of IoMT technology, time and effort can be saved for both patients and doctors. The burden on healthcare systems can be decreased by IoMT, through the secure communication that links patients to their doctors [26]. The swift utilization of IoMT is expected to initiate the development of various frameworks that can rapidly and precisely diagnose the health of patients and heal various illnesses remotely and in a secure manner. There exist a considerable number of applications based on IoMT, especially for illnesses that threaten the lives of patients, such as COVID-19 [27] and heart failure [28].

In this paper, a new medical IoT-based framework is proposed, which aids hematologists by automatically diagnosing microscopic images of acute leukemia patients. The main contributions of our study are as follows:

- Showing the feasibility of applying our IoT-based diagnosis systems for cancer patients, saving leukemia test times and requiring minimal hardware resources at the clinical laboratories.
- Diagnosing acute leukemia diseases for COVID-19 patients can be done in a safe clinical environment using our proposed medical IoT framwork.
- Developing a new generative adversarial network (GAN) classifier to handle a small image data set of blood cells without using data augmentation and/or transfer learning techniques.
- Conducting comparative evaluation between our developed GAN model with other deep classification models, in order to demonstrate the superior performance of our IoT-based framework when identifying cancer blood cases.

The remainder of this paper is structured as follows. Section 2 provides a review of related research works focused on automated classification of leukemia images using machine and deep learning techniques in medical IoT environments. Section 3 describes the proposed medical IoT-based framework, including our developed microscopic blood image classifier. The results and evaluation of extensive experiments are presented in Section 5. A discussion and our conclusions, along with future directions of study, are given in Section 5 and Section 6, respectively.

## 2. Related Works

Numerous IoMT CAD systems have been proposed in previous studies for the diagnosis of leukemia. These studies have utilized different machine learning and deep learning models for early detection of leukemia and its sub-classes. Mohamed et al. [29] have adopted a random forest (RF) ensemble method to detect WBC cancers. The proposed method achieved an acceptable accuracy of 94.3%. The K-means clustering algorithm has been exploited for the detection of ALL [30]. The proposed model achieved an accuracy score of 92.8%; however, the model was only trained and validated on a small number of samples (i.e., 60 samples).

Sharma and Kumar [31] have presented a modified version of principal component analysis (PCA) to reduce the number of features and combined the artificial bee colony (ABC) algorithm and a back-propagation neural network (BPNN) to differentiate leukemia cells from each other. The proposed technique achieved good average accuracy (i.e., 98.72%) and computation time. Jothi et al. [32] have conducted a comparative study evaluating the detection performance for leukemia diseases using various machine learning algorithms, such as Jaya, naïve Bayes (NB), support vector machine (SVM), linear discriminant analysis (LDA), and decision tree (DT). The authors first segmented the blood images using a clustering technique known as the backtracking search algorithm (BSA). The performance, in terms of accuracy, was better when combining Jaya and SVM or DT, compared to the other techniques. Huang et al. [4] have investigated the effectiveness of utilizing the bone marrow cell microscopy images in the diagnosis of three leukemia sub-classes (i.e., AML, CML, and ALL). The proposed framework adopted both transfer learning and a CNN for the early detection of the leukemia sub-classes. The authors collected their own dataset, consisting of 104 bone marrow smears (18 subjects were healthy, 53 were AML patients, 18 were CML patients, and 23 were ALL patients). The authors first utilized two algorithms for pre-processing purposes: A self-adaptive filter algorithm and the perfect reflection algorithm. Thereafter, they used three CNN architectures (i.e., ResNet-50, Inception-V3, and DenseNet121) to classify the data generated in the pre-processing step. Their experimental results showed that DenseNet121 outperformed the other methods, in terms of classification accuracy (its accuracy is 74.8%). The authors then conducted another experiment to evaluate the performance of the algorithms after pre-training the models using transfer learning. DenseNet121 outperformed the other algorithms again, yielding a prediction accuracy of 95.3%.

Bibi et al. [33] have proposed an IoMT-based residual convolutional neural network (ResNet-34) and dense convolutional neural network (DenseNet-121) for leukemia sub-class classification. They conducted experiments involving the detection of healthy vs. leukemia sub-class patients, and showed that the proposed framework could outperform some famous traditional machine learning algorithms. However, their results were misleading, as the data augmentation technique was applied to both training and testing samples, in order to handle the small number of microscopic images. Consequently, the accuracy scores of the proposed ResNet-34 and DenseNet-121 models on augmented or synthetic tested images were not realistic.

Ahmed et al. [34] have presented an automated deep model to classify leukemia and healthy blood microscopic images using a CNN-based approach. Due to the limited number of training samples, the authors applied seven data augmentation techniques to increase the number of training instances. To prove the effectiveness of their method, it was compared with other machine learning algorithms. The two conducted experiments demonstrated the effectiveness of the proposed method, in terms of accuracy, in comparison with the other algorithms. The data set was divided into two classes—healthy and leukemia—in the first experiment, and into five classes in the second experiment (i.e., the four sub-classes and the healthy class). The resulting accuracy scores were 88.25% in the first experiment and 81.74% in the second experiment. The proposed model proved its effectiveness and achieved high accuracy in binary classification of the two classes: Healthy vs. leukemia.

## 3. Methods

### 3.1. Microscopic Blood Data Set

**Figure 1:**
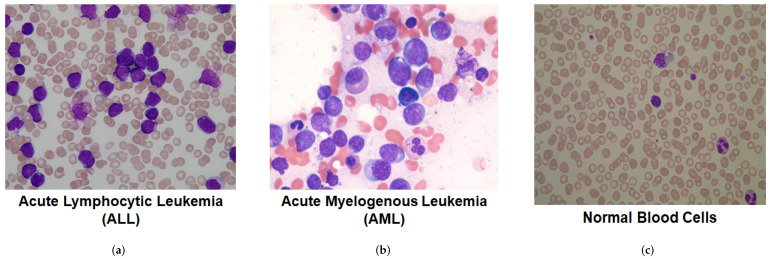
Three different samples from microscopic blood data set, representing: (**a**) Acute lymphocytic leukemia; (**b**) Acute myelogenous leukemia; and (**c**) Normal blood cells.

In this study, all microscopic blood cell images with acute leukemia diseases were selected, including three classes: ALL, AML and normal blood smears (see Figure 1). The data set was collected from two different public sources, ALL-IDB [35] and the American Society of Hematology (ASH) image bank [36]. The ALL-IDB data set provides annotated microscopic images of blood cells for ALL types of leukemia and normal cases only. It was established by experienced oncologists for classification and segmentation tasks, as well as for the evaluation of new relevant machine learning and deep learning algorithms in the field. Images of the AML type of leukemia were provided the through freely available ASH image bank, which aims to support various hematological research subjects. Table 1 illustrates the total number of microscopic blood images (i.e., 445 images for all conditions of healthy and blood cancers).

### 3.2. Generative Adversarial Networks

Goodfellow et al. [37] developed the GAN model, which has recently become an increasingly attractive topic for AI researchers and experts. GANs have shown effective performance as a major class of deep neural networks, due to their advantageous built-in capabilities to generate synthetic images, instead of using data augmentation techniques [38,39]. This has allowed GANs to successfully handle the training phase of proposed deep network models while using small data sets, especially for medical applications such as COVID-19 detection [40], or biomedical image enhancement and segmentation [41,42].

**Figure 2:**
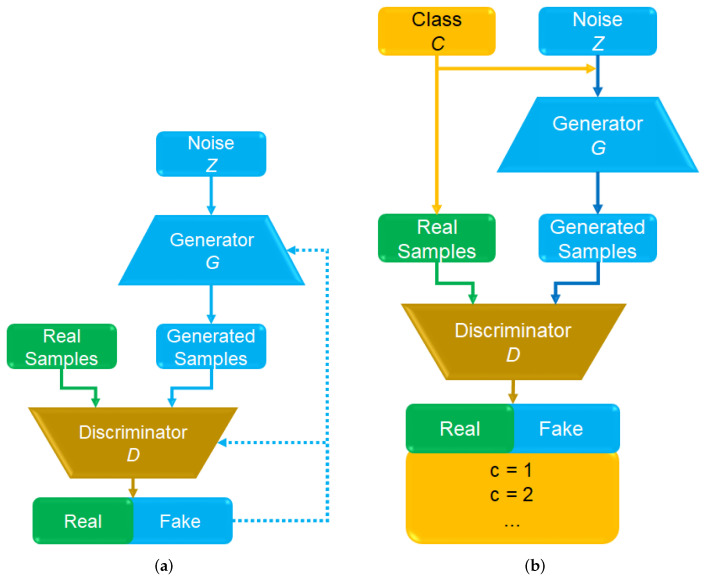
(**a**) Basic structures of the GAN model; and (**b**) the GAN with auxiliary classifier.

Figure 2 depicts a basic GAN model including two different networks, named the generator and discriminator [37]. Training of these networks is carried out simultaneously. The generator is responsible for producing synthetic or fake images, while the discriminator performs binary classification of real and fake images [43]. The probability of both real images from the data set and fake images from the generator *G* are estimated by the discriminator *D*. Hence, the training procedure of a GAN model can be considered as min-max competitive learning between the networks *G* and *D*, as described in Equation (1), where *z* is a random noise, the real and generated data distributions are $p_{data}$ and $p_{z}$, respectively, G(z) is the noisy sample output of the generator, and D(x) represents the probability value of the discriminator for a real sample *x* [44], where D(x)=1 in the case where the input data source is real, and D(G(z))=0 for a fake image produced by G(z). Maximizing the training accuracy of the discriminator *D* is important for achieving the iterative binary classification procedure [45].

(1) $$\min_{G}\max_{D}=E_{x∼P_{data}}\left(x\right)\left[logD\left(x\right)+E_{z∼P_{z}\left(x\right)}\left[log\left(1-D\left(G\left(z\right)\right)\right)\right]\right].$$

The Auxiliary classifier with GAN (AC-GAN) model [46] is the focus of this study, which is shown in Figure 2b. The traditional AC-GAN method was mainly developed for the creation of synthetic images with high resolution in an unsupervised learning manner. However, we consider the semi-supervised classification of the AC-GAN model, in order to accomplish the accurate identification of microscopic blood images. Hence, the class labels *C* of real images were also used to train the discriminator *D*. In addition to the binary classification results of the discriminator, the expected classes of real samples are also linked with the *D* outputs, as depicted in Figure 2a.

### 3.3. Proposed Blood Diagnosis System

**Figure 3:**
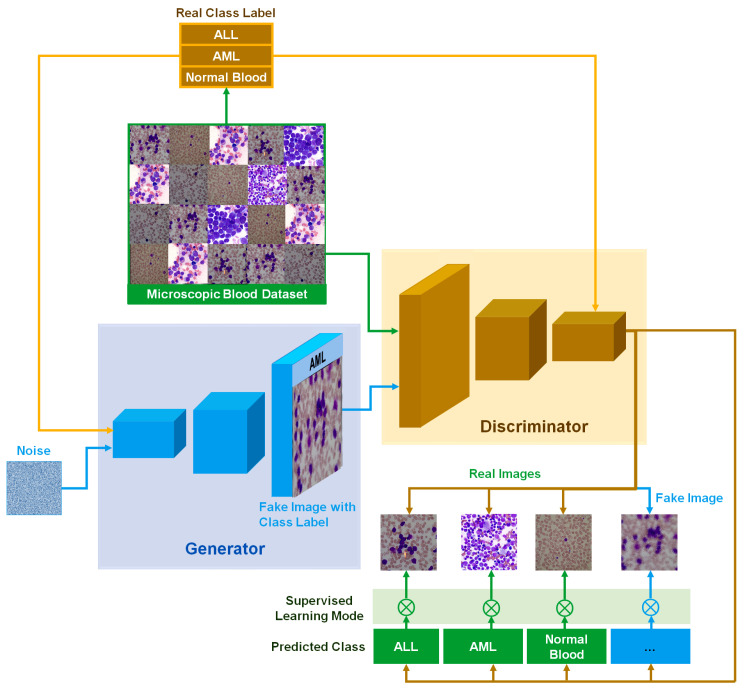
Workflow of our developed GAN classifier for identifying acute leukemias and normal cases from microscopic blood images.

Our developed AC-GAN classifier is similar to the basic AC-GAN, using conditional class labels in order to produce fake images of acceptable resolution. The role of the auxiliary classifier is still to predict the real class labels, integrated with the real and fake image classification of the discriminator *D*. We designed our AC-GAN model to include both semi-supervised and unsupervised learning modes, to achieve the classification of real blood images linked with real class labels, as shown in Figure 3. The developed AC-GAN assigns a class label to each generated image, c∼pc. In this scenario, the fake images are generated by adding the noise *z* to the output of generator G:Xfake=G(c,z). As shown in Equations (2) and (3), the objective function $V_{acgan}$ includes the log-likehood of the correct source, $L_{s}$, and the correct class, $L_{c}$, where the training of *G* aims to minimize the difference $\left(L_{c}-L_{s}\right)$. In contrast, the goal of *D* is to maximize the sum $\left(L_{s}+L_{c}\right)$ [46].

(2) $$V_{acgan}\left(G,D\right)=L_{s}+L_{c},$$

(3) $$L_{s}=E\left[logP\left(S=real|X_{real}\right)\right]+E\left[logP\left(S=fake|X_{fake}\right)\right],$$

(4) $$L_{c}=E\left[logP\left(C=c|X_{real}\right)\right]+E\left[logP\left(C=c|X_{fake}\right)\right].$$

Figure 3 shows that our developed AC-GAN performs the same operations as a basic GAN. Binary classification is used to identify whether the microscopic image is real or not. Then, utilizing the unsupervised learning mode, the output of the auxiliary classifier predicts the class label matching the corresponding real blood image only. As a result, in supervised learning mode, we included the operator (⊗) as a switch to handle the output of *D*, in order to conduct the link between real microscopic images and the true predicted class labels [47]. This eliminates the need to develop extra samples for all classes in this study, by using the same discriminator and generator, resulting in the effective identification of ALL and AML diseases for all tested blood images. We did not consider the class labels of fake images, as the generation of high-resolution synthetic microscopic images was not the goal of this study.

**Figure 4:**
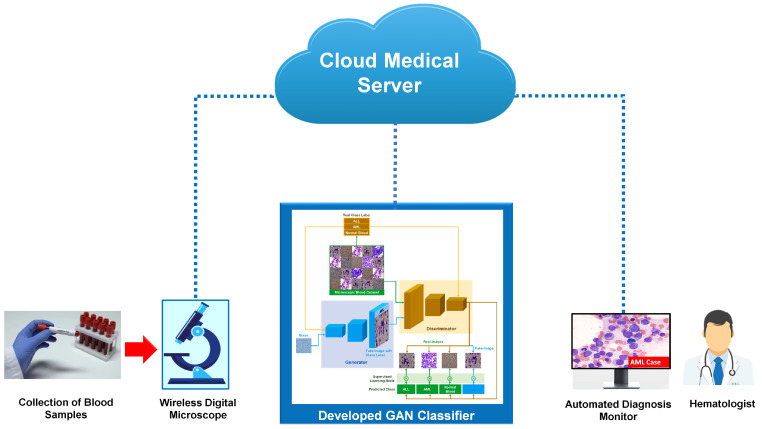
Schematic diagram of our proposed medical IoT-based diagnosis framework for automatic identification of the blood conditions of patients using wireless microscopic imaging of samples and the developed GAN classifier.

The overall framework of our proposed IoMT-based microscopic blood image diagnosis method is depicted in Figure 4. It is composed of three main stages, as follows: First, the blood samples are taken from patients and collected for leukemia tests. Based on a wireless microscopic imaging system, the blood smear images are sent to cloud medical server to provide further options. For instance, the blood samples, including dates and results, can be automatically recorded in patient medical files. Second, the acquired blood images are analyzed using our developed AC-GAN classifier, as shown in Figure 3. In our proposed framework, utilizing cloud computing services is highly recommended for the automated classification of all uploaded microscopic images, in order to lessen the required hardware resources and storage; for example, reducing the need for Graphical Processing Units (GPUs) and large memory capacities. Third, the blood diagnosis results are sent to the hematologist’s monitor or smartphone, in order to verify and finalize the blood analysis report with medical recommendations.

### 3.4. Performance Analysis of GAN Classifier

**Figure 5:**
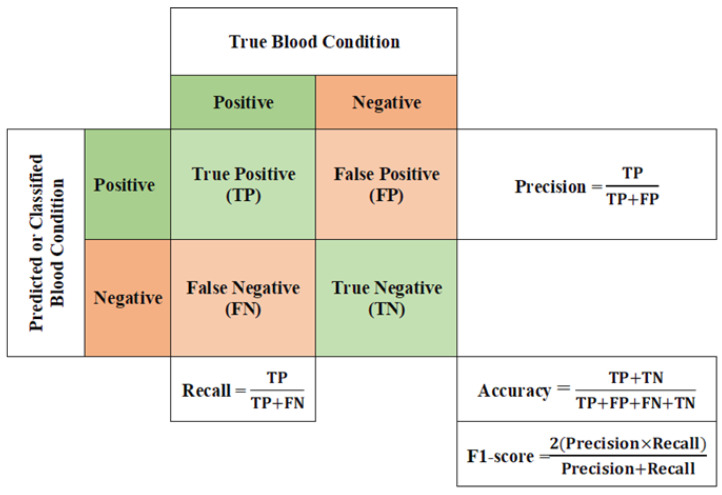
A confusion matrix and evaluation metrics for the microscopic blood image classification results presented in this study.

The following metrics were used to assess the performance of our AC-GAN model for classification of acute leukemias in microscopic images, based on cross-validation estimation [48]: a confusion matrix and four evaluation metrics were used, as shown in Figure 5. True positive (TP), false positive (FP), false negative (FN), and true negative (TN) are the expected outputs of the confusion matrix. The diagnosis results of hypothesis testing for each anticipated class, with respect to its true class, are reflected in these results. Accuracy is the essential metric for most image-based classifiers. It is calculated by dividing the sum of true positives (TP) and true negatives (TN) by all possible cases, as shown in Figure 5. The accuracy is usually presented as a percentage (e.g., 100%). The precision is used to describe the relationship between real positive predicted values and all positive predicted values. The recall or sensitivity gives the ratio between the predicted TP value and the sum of predicted TP and FN values. The F1-score is the fourth evaluation metric, which includes the double ratio of recall and precision metrics. In addition, the classification performance of our developed GAN model was compared with those of existing transfer learning models, including DenseNet-121 [49], ResNet-50 [50], and VGG-16 [51]. Moreover, a comparison with other deep learning models from previous studies was also carried out, in order to verify the findings of this study, as presented in the following section.

## 4. Experiments

The developed GAN classifier and other deep network models were programmed using the Scientific Python Development Environment (Spyder 5.1.5) and the Tensorflow deep learning package (Keras 2.7) [52]. We conducted all tests and blood classification evaluations on a laptop with Core (TM) i7-2.2 GHz processor, 4 GB NVIDIA GPU, and 16 GB RAM. The blood image data set was represented in RGB color format. Each microscopic image was scaled to 28 × 28 pixels, to make them suitable for our computing resources and the developed GAN classifier, while maintaining good quality for all tested images.

Two main experiments were conducted to evaluate the classification performance of our GAN model. First, the binary classification of ALL against normal blood cases was carried out, using only the ALL-IDB data set. Second, multi-class classification of three blood conditions—namely, ALL, AML, and normal blood cells—was carried out, based on the combination of ALL-IDB and ASH image data sets. To start the training phase of the deep network models, all microscopic images of ALL, AML, and normal blood cells (see Table 1) were randomly split in a 80:20 percent ratio, where the validation and testing phases utilized 20% of the blood images (i.e., 74 of 368 images for binary classification and 89 of 445 images for multi-class classification tests). The hyperparameters were manually tuned for our developed GAN classifier, where the learning rate, the batch size, and the number of epochs were $10^{-3}$, 64, and 50, respectively. Furthermore, the Adam stochastic optimization method [53] was exploited to accomplish the training phase of all classifiers. The Softmax activation function was used in the classifier output layer, in order to predict leukemia and normal classes for all tested microscopic blood smear samples.

### 4.1. Acute Leukemia Classification Results

**Figure 6:**
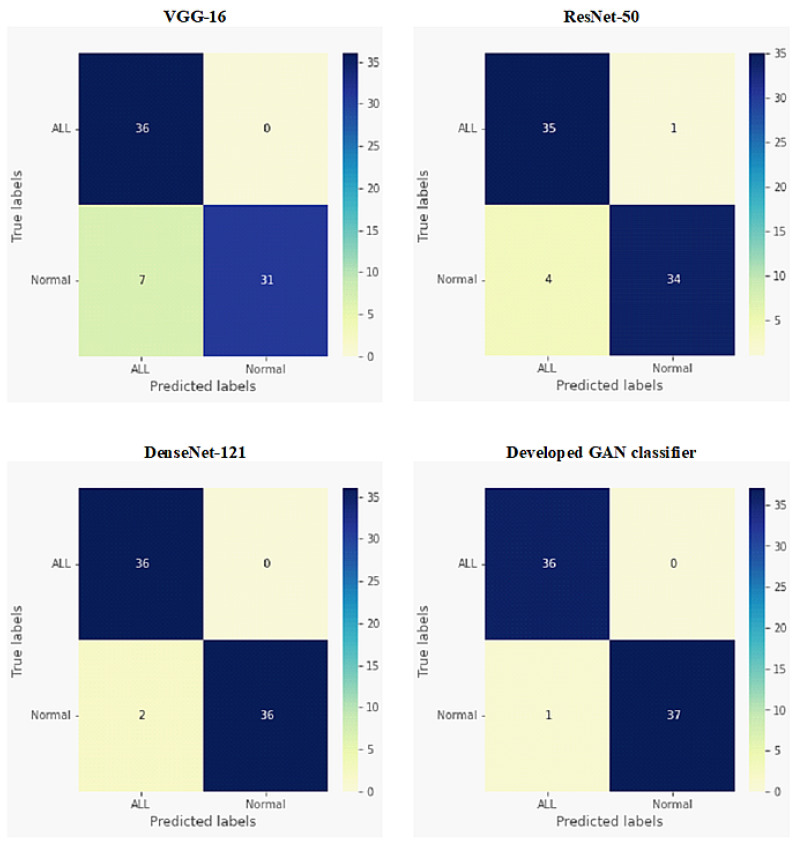
Confusion matrices for binary classification of ALL disease versus normal cases for all tested deep network models.

**Figure 7:**
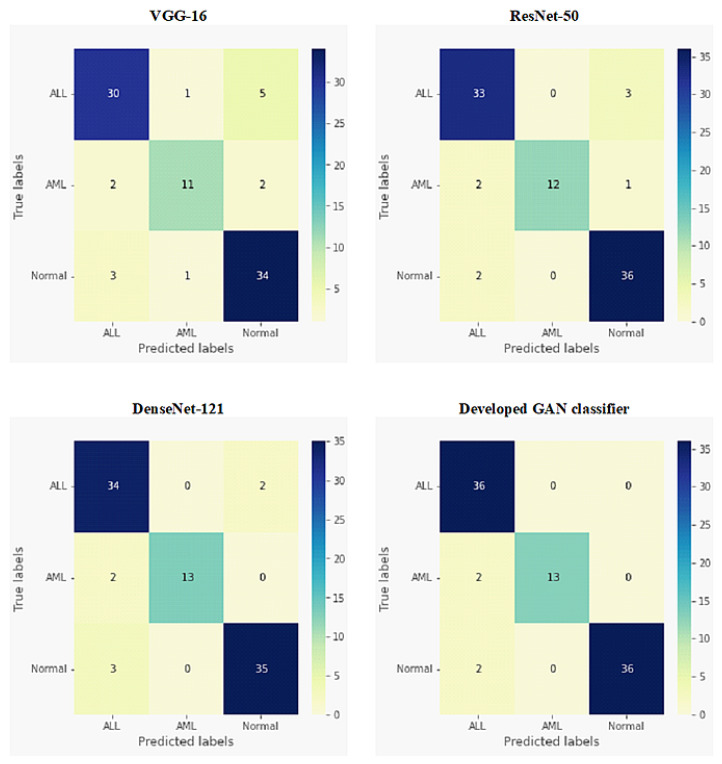
Confusion matrices for multi-class classification of ALL, AML, and normal blood cells for all tested deep network models.

The confusion matrices for both binary classification and multi-class classification of ALL, AML, and normal blood conditions are depicted in Figure 6 and Figure 7, respectively. These results were achieved by our developed GAN classifier and three transfer learning models: VGG-16, ResNet-50, and DenseNet-121. For the binary classification (ALL or normal) results shown in Figure 6, the developed GAN model showed the highest accuracy score, with one misclassified normal case sample. Similarly, DenseNet-121 achieved accurate results, but had two misclassified images for the normal class. ResNet-50 and VGG-16 showed moderate and worse performances, respectively, when identifying ALL and normal blood conditions. As shown in Figure 7, the developed GAN presented the best accuracy for the three-class (ALL, AML and normal blood) classification, with four misclassified samples (i.e., two AML images and two normal case images). The VGG-16 model failed to achieve the multi-class classification task precisely.

As detailed in Figure 5, the four evaluation metrics of precision, recall, F1-score, and accuracy are illustrated in Table 2 and Table 3, for all tested classifiers in the binary and multi-class experiments, respectively. For binary classification, as shown in Table 2, the developed GAN classifier and DenseNet-121 achieved the best accuracy scores, above 97%. Although the VGG-16 model completely succeeded in identifying ALL cases, it could not classify normal blood images accurately, thus presenting the lowest accuracy score of 90.54%. The developed GAN classifier was capable of achieving the best values for all evaluation metrics, with an accuracy score of 95.50%, for the multi-class classification task, as shown in Table 3. DenseNet-121 presented the second-best classification results for leukemia diseases, achieving an accuracy of 92.13%. ResNet-50 achieved an accuracy score of 91.01%, indicating moderate classification performance for all tested blood smear images.

### 4.2. Comparison with Previous Studies

Table 4 illustrates the relative characteristics of our developed GAN classifier, when compared to other machine learning and deep learning models used in previous studies focused on automated leukemia diagnosis. Most related works have conducted binary classification of microscopic blood smears using the ALL-IDB data set [35]; for instance, a CNN [34] and VGG-16 [54] have been applied to identify ALL or normal cases, with corresponding accuracy scores of 88.25% and 96.84%, respectively. Furthermore, a machine learning technique, named SVM [55], has been utilized and achieved an accuracy score of 98.0% for AML versus healthy blood smears, based on the ASH image bank [36]. A combination of the ALL-IDB data set and ASH image bank has been carried out to perform multi-class classification of leukemia diseases, as presented in [8,33]. In addition, a private blood data set has been collected from different hospitals for testing the classification of acute and chronic leukemias (ALL, AML, and CML) using a fine-tuned DenseNet-121, which achieved an accuracy score of 95.30% [5]. Combined machine learning and deep learning models (e.g., SVM with DensNet-121 or ResNet-50) have been exploited to obtain maximal accuracy values of 98.0% for binary classification and 96.67% for multi-class classification, as illustrated in Table 4. Nevertheless, our developed GAN classifier showed the best accuracy scores: higher than 95.5% for all tested cases when using public microscopic blood image data sets.

## 5. Discussion

The development of Intelligent IoT-based systems has become a recent trend for advanced medical procedures, and for the image-guided diagnosis of acute blood cancers in particular. Microscopic blood smear testing is the gold standard of leukemia tests, which may be analyzed through wireless digital microscopy. Therefore, the automated diagnosis of acute blood cancer diseases in this study was successfully achieved using our developed GAN classifier integrated within an IoMT framework, as shown in Figure 3 and Figure 4. As detailed in Table 1, the public image data sets of ALL-IDB [35] and ASH image bank [36] were used as benchmark data for validating the classification performance of our GAN model, in terms of accomplishing diagnostic procedures for acute leukemia patients. Compared to deep network models, such as VGG-16, ResNet-50, and DenseNet-121, the evaluation results for acute leukemia classification demonstrated the competitive performance of developed GAN classifier, in terms of achieving the highest accuracy scores for binary classification on ALL or healthy blood images and multi-class classification on ALL, AML, and normal blood images, as illustrated in Table 2 and Table 3, respectively.

A semi-supervised AC-GAN model has been developed to accomplish the automatic multi-task classification of acute leukemias from microscopic blood images, as depicted in Figure 3. In the identification of ALL disease against normal blood cases, the developed GAN classifier showed the best binary classification results among other deep transfer learning models (i.e., VGG-16, ResNet-50, and DenseNet-121), as presented in Figure 6 and Table 2. Similarly, the superior performance of our developed GAN was also achieved when carrying out multi-class classification of acute leukemias, showing the highest accuracy score of 95.50%, as presented in Figure 7 and Table 3. DenseNet-121 also showed good results for all classification tests of leukemia smears, and was relatively equal to our developed GAN classifier. However, the main advantage of GAN approaches over deep transfer learning models is as follows. A small number of microscopic blood images was publicly available for training and testing the proposed classifiers, as illustrated in Table 1. In this case, the training data are insufficient to achieve the expected performance of models such as DenseNet-121, and data augmentation techniques must be applied to solve this problem [56]. To the contrary, the developed GAN model can self-generate additional good-quality fake images to improve the blood image classification training procedure. Furthermore, unsupervised and semi-supervised learning techniques, such as GANs, are more powerful than supervised models in medical applications, as the creation of a fully annotated data set is usually a tedious and time-consuming task for medical staff.

Table 4 illustrates the comparative characteristics between our GAN model and other machine learning and deep learning models in relevant research works, which verifies the effective performance of the developed classifier when using the same microscopic blood data. For both binary and multi-class leukemia sub-type classification, transfer learning classifiers (e.g., VGG-16 and DenseNet-121) showed good results. In addition, the SVM algorithm integrated with DenseNet-121 and ResNet-50 [8] provided high accuracy scores of 98.0% and 96.67% for binary and multi-class classification tasks, respectively. Our developed GAN classifier outperformed this model when identifying ALL against healthy cases, but gave a slightly lower accuracy (of 95.58%) than the ResNet-50 + SVM classifier (with an accuracy of 96.67%), as reported in Table 4. Nevertheless, the ResNet-50 + SVM model was evaluated not only on the ALL-IDB data set, but also on a heterogenous data set to achieve this superior classification result [8].

The high computational resources required, such as GPUs and large memory size, is a main drawback of deep network models such as DenseNet-121 and ResNet-50. The developed GAN classifier generates synthetic data during the training phase, which also leads to a high storage capacity requirement. Nevertheless, this hardware resource requirement can be fulfilled by utilizing cloud computing services in our proposed IoMT framework, as depicted in Figure 4. In addition, all hyperparameter values of our GAN classifier and implemented deep network models were manually tuned in this study. This manual tuning procedure is an iterative and time-consuming task, in order to eventually obtain good classification results. Therefore, neural architecture search methods [57] will be used in our future studies, in order to automate the design of our developed GAN classifier. Security and privacy aspects of patient data and leukemia diagnosis results will be also considered in our proposed medical IoT-based system, to be adopted for open communications and networked computing systems. However, the proposed IoMT-system including our developed GAN classifier is still valid to successfully achieve the automated diagnosis of acute leukemia diseases.

## 6. Conclusions and Future Research Directions

In this article, we presented a new medical IoT framework for the automated diagnosis of acute leukemia sub-classes, namely, the ALL and AML diseases. The proposed IoMT framework utilizes cloud computing services to provide accurate online leukemia tests, saving hematological efforts and lowering the required computing resources. An advanced deep learning architecture, the AC-GAN model, was developed to identify leukemia and its two sub-classes. Two publicly available data sets of microscopic blood images were used to evaluate the effectiveness of the developed GAN classifier. Compared with previous works, our semi-supervised AC-GAN model showed promising classification results for acute leukemias, as illustrated in Table 4. In the future, we plan to add more samples and sub-classes of acute and chronic blood cancers. Automating the design of the developed GAN model comprises our next research milestone in order to streamline the method while enhancing the classification performance. Furthermore, an implementation of our proposed medical IoT framework in the clinical routine of leukemia tests should be realized in order to support both hematologists and cancer patients, especially in the context of the COVID-19 pandemic.

## Figures and Tables

**Table 1:** Summary of microscopic image data sets for the different blood conditions considered in this study.

Condition of Blood Cells	Data Set	Number of Images
ALL	ALL-IDB	179
AML	ASH Image Bank	77
Normal	ALL-IDB	189
Total		445

**Table 2:** Evaluation metrics for all tested binary classifiers on microscopic blood images.

Classification Model	Class	Precision	Recall (Sensitivity)	F1-Score	Accuracy
VGG-16	ALL	0.84	**1.00**	0.91	0.9054
	Normal	**1.00**	0.82	0.90	
ResNet-50	ALL	0.90	0.97	0.93	0.9324
	Normal	0.97	0.89	0.93	
DenseNet-121	ALL	0.95	**1.00**	0.97	0.9730
	Normal	**1.00**	0.95	0.97	
Developed GAN Classifier	ALL	**0.97**	**1.00**	**0.99**	**0.9865**
	Normal	**1.00**	**0.97**	**0.99**	

**Table 3:** Evaluation metrics for all tested multi-class classifiers on microscopic blood images.

Classification Model	Class	Precision	Recall (Sensitivity)	F1-Score	Accuracy
VGG-16	ALL	0.86	0.83	0.85	0.8430
	AML	0.85	0.73	0.79	
	Normal	0.83	0.89	0.86	
ResNet-50	ALL	0.89	0.92	0.90	0.9101
	AML	**1.00**	0.80	0.89	
	Normal	0.90	**0.95**	0.92	
DenseNet-121	ALL	0.87	0.94	0.91	0.9213
	AML	**1.00**	**0.87**	**0.93**	
	Normal	0.95	0.92	0.93	
Developed GAN Classifier	ALL	**0.90**	**1.00**	**0.95**	**0.9550**
	AML	**1.00**	**0.87**	**0.93**	
	Normal	**1.00**	**0.95**	**0.97**	

**Table 4:** Comparison between our developed GAN and other models in previous studies for the classification of leukemias.

Classification Model	Tested Data Set	Classification Task	Accuracy (%)
CNN [34]	ALL-IDB and ASH image bank	Binary (ALL vs. normal)	88.25
		Multi-class (acute and chronic leukemia sub-types)	81.74
SVM [55]	ASH image bank	Binary (AML vs. normal)	98.00
VGG-16 [54]	ALL-IDB	Binary (ALL vs. normal)	96.84
DenseNet-121 [4]	Private Dataset from Guangdong Second Provincial General	Multi-Class (ALL, AML, CML, and Normal)	95.30
	Hospital, and Zhujiang Hospital of Southern Medical University		
DenseNet-121 with SVM ResNet-50 with SVM [8]	Mixed data set including ALL-IDB	Binary (ALL vs. Normal)	98.00
	images	Multi-class (ALL, AML, and Normal)	**96.67**
Developed GAN Classifier	ALL-IDB and ASH image bank	Binary (ALL vs. Normal)	**98.65**
		Multi-class (ALL, AML, and Normal)	95.58

## Data Availability

The data that support the findings of this research are publicly available, as indicated in the references.
